# Supplementary material for: The molecular basis of drug selectivity for α5 subunit-containing GABAA receptors
Source: Nat Struct Mol Biol. 2023 Oct 30;30(12):1936–46. doi: 10.1038/s41594-023-01133-1 (PMC10716045; doi:10.1038/s41594-023-01133-1)
Supplement: Supplementary file 1 — Reporting Summary [file 41594_2023_1133_MOESM1_ESM.pdf]

Corresponding author(s): Paul S. Miller
Maria-Clemencia Hernandez

Last updated by author(s): Feb 28, 2023

Reporting Summary

Nature Portfolio wishes to improve the reproducibility of the work that we publish. This form provides structure for consistency and transparency in reporting. For further information on Nature Portfolio policies, see our Editorial Policies and the Editorial Policy Checklist.

Statistics

For all statistical analyses, confirm that the following items are present in the figure legend, table legend, main text, or Methods section.

n/a	Confirmed	
☐	☒	The exact sample size (*n*) for each experimental group/condition, given as a discrete number and unit of measurement
☐	☒	A statement on whether measurements were taken from distinct samples or whether the same sample was measured repeatedly
☒	☐	The statistical test(s) used AND whether they are one- or two-sided<br>*Only common tests should be described solely by name; describe more complex techniques in the Methods section.*
☒	☐	A description of all covariates tested
☒	☐	A description of any assumptions or corrections, such as tests of normality and adjustment for multiple comparisons
☐	☒	A full description of the statistical parameters including central tendency (e.g. means) or other basic estimates (e.g. regression coefficient) AND variation (e.g. standard deviation) or associated estimates of uncertainty (e.g. confidence intervals)
☒	☐	For null hypothesis testing, the test statistic (e.g. *F*, *t*, *r*) with confidence intervals, effect sizes, degrees of freedom and *P* value noted<br>*Give P values as exact values whenever suitable.*
☒	☐	For Bayesian analysis, information on the choice of priors and Markov chain Monte Carlo settings
☒	☐	For hierarchical and complex designs, identification of the appropriate level for tests and full reporting of outcomes
☒	☐	Estimates of effect sizes (e.g. Cohen's *d*, Pearson's *r*), indicating how they were calculated

Our web collection on statistics for biologists contains articles on many of the points above.

Software and code

Policy information about availability of computer code

Data collection	FEI EPU v2.13, HKL2000
Data analysis	Warp 1.09, cryoSPARC v3.2.0, relion 4.0, UCSF Chimera v1.13.1, Pymol v2.1.1, Coot 0.9.3, Phenix 1.19.1, MolProbity, HOLE, Graphpad Prism 9, OriginPro2015, Ionflux16Analysis, Star Aniso, Autoproc-Aimless, Phaser v2.7, Buster TNT, Refmac v5.7, Grade Server.

For manuscripts utilizing custom algorithms or software that are central to the research but not yet described in published literature, software must be made available to editors and reviewers. We strongly encourage code deposition in a community repository (e.g. GitHub). See the Nature Portfolio guidelines for submitting code & software for further information.

Data

Policy information about availability of data

All manuscripts must include a data availability statement. This statement should provide the following information, where applicable:

- Accession codes, unique identifiers, or web links for publicly available datasets
- A description of any restrictions on data availability
- For clinical datasets or third party data, please ensure that the statement adheres to our policy

Atomic models were built using the homology source model PDB-4COF from the Protein Data Bank. Atomic model coordinates have been deposited in the Protein Data Bank, and Cryo-EM maps have been deposited in the Electron Microscopy Data Bank, as follows: a5V1-Flumazenil, PDB8BGI; a5V2-Bretazenil, PDB-8BHG; a5V3-apo, PDB-8BEJ, EMD-16005; a5V3-Diazepam, PDB-8BHK, EMD-16058; a5V3-DMCM, PDB-8BHM, EMD-16060; a5V3-RO154513, PDB-8BHB, EMD-16051; a5V3-

RO4938581, PDB-8BHS, EMD-16068; a5V3-L655,708, PDB-8BHO, EMD-16063; a5V3-Basmisanil, PDB-8BHA, EMD-16050; a5V3-RO7015738, PDB-8BHR, EMD-16067; a5V3-RO7172670, PDB-8BHQ, EMD-16066; a5V3-RO5211223, PDB-8BHI, EMD-16055.

Human research participants

Policy information about studies involving human research participants and Sex and Gender in Research.

Reporting on sex and gender	N/A
Population characteristics	N/A
Recruitment	N/A
Ethics oversight	N/A

Note that full information on the approval of the study protocol must also be provided in the manuscript.

Field-specific reporting

Please select the one below that is the best fit for your research. If you are not sure, read the appropriate sections before making your selection.

☒ Life sciences ☐ Behavioural & social sciences ☐ Ecological, evolutionary & environmental sciences

For a reference copy of the document with all sections, see nature.com/documents/nr-reporting-summary-flat.pdf

Life sciences study design

All studies must disclose on these points even when the disclosure is negative.

Sample size	No statistical methods were used to estimate appropriate sample size. Single structures arise from thousands or millions of copies of particles, with resolution determined by number and quality of particles input. For radioligand binding and electrophysiology experiments 3-10 biological repeats were performed based on experience and standard practice in the field for these kinds of experiments. Because differences are typically large relative to error, small sample sizes are sufficient.
Data exclusions	Following standard cryoSPARC and relion processing pathways, best representative 2D and 3D classes were selected for the final particle reconstructions. Poor quality and irrelevant other classes were discarded. There were no radioligand or electrophysiology data exclusions.
Replication	Radioligand experiments were organised to be repeated on separate days, i.e. to be separate experiments, not technical repeats in the same plate, and were repeated in this manner at least 3 times or more. All attempts at replication were successful. One cryo-EM structure was generated from each independent dataset of thousands or millions of particles. The protein structure was generated 8 times from three separate rounds of protein production and purification, and protein structures were consistent across batches. For each single structure two independent maps of each cryo-EM sample were generated in order to estimate resolution according to the recommended procedures in the field (the 'gold standard').
Randomization	Because all samples used in this study were indistinguishable from each other, whether it be protein batches for cryo-EM, or membrane preps for radioligand binding, or cells for electrophysiology, randomization was not relevant to this study. I.e. the study did not involve participants with different features and properties, but instead homogeneous sample sets of protein, membranes or cells.
Blinding	Blinding was not relevant to this study because datasets were processed by computer software, minimizing the possibility of experimenter bias.

Reporting for specific materials, systems and methods

We require information from authors about some types of materials, experimental systems and methods used in many studies. Here, indicate whether each material, system or method listed is relevant to your study. If you are not sure if a list item applies to your research, read the appropriate section before selecting a response.

Materials & experimental systems

n/a	Involved in the study
☐	☒ Antibodies
☐	☒ Eukaryotic cell lines
☒	☐ Palaeontology and archaeology
☒	☐ Animals and other organisms
☒	☐ Clinical data
☒	☐ Dual use research of concern

Methods

n/a	Involved in the study
☒	☐ ChIP-seq
☒	☐ Flow cytometry
☒	☐ MRI-based neuroimaging

Antibodies

Antibodies used

Rho-1D4 antibody was purchased from the University of British Columbia (https://ubc.flintbox.com/technologies/0f1ef64b-fa5d-4a58-9003-3e01f6f672a6). 50 mg of 1D4 antibody in 20 ml phosphate buffered saline was coupled to 3.3 g dry powdered cyanogen bromide beads (see methods).. The megabody MbF3 was obtained from the laboratory of Professor Jan Steyeart (VUB).

Validation

The Rho-1D4 antibody was previously validated to bind the sequence TETSQVAPA (PMID: 6188482). We have previously validated it can be used to affinity purify GABA-A receptors with the TETSQVAPA C-terminal tag (PMID: 24909990). The megabody MbF3 was characterised and published elsewhere (PMID: 33408403).

Eukaryotic cell lines

Policy information about cell lines and Sex and Gender in Research

Cell line source(s)

HEK293T cells, HEK293F cells, and HEK 293S GnTI- cells were obtained from ATCC.

Authentication

Further authentication was not performed for this study.

Mycoplasma contamination

Mycoplasma testing was not performed for this study.

Commonly misidentified lines
(See ICLAC register)

No commonly misidentified cell lines were used in this study.
